# An attempt to identify brain tumour tissue in neurosurgery by mechanical indentation measurements

**DOI:** 10.1007/s00701-024-06218-4

**Published:** 2024-08-21

**Authors:** Isabelle Skambath, Jessica Kren, Patrick Kuppler, Steffen Buschschlueter, Matteo Mario Bonsanto

**Affiliations:** 1 Department of Neurosurgery, UKSH, University of Luebeck, Luebeck, Germany; 2 Soering GmbH, Quickborn, Germany

**Keywords:** Tissue biomechanics, Brain tumour surgery, Indentation measurements, Tissue classification, Elasticity

## Abstract

**Background:**

The intraoperative differentiation between tumour tissue, healthy brain tissue, and any sensitive structure of the central nervous system is carried out in modern neurosurgery using various multimodal technologies such as neuronavigation, fluorescent dyes, intraoperative ultrasound or the use of intraoperative MRI, but also the haptic experience of the neurosurgeon. Supporting the surgeon by developing instruments with integrated haptics could provide a further objective dimension in the intraoperative recognition of healthy and diseased tissue.

**Methods:**

In this study, we describe intraoperative mechanical indentation measurements of human brain tissue samples of different tumours taken during neurosurgical operation and measured directly in the operating theatre, in a time frame of maximum five minutes. We present an overview of the Young’s modulus for the different brain tumour entities and potentially differentiation between them.

**Results:**

We examined 238 samples of 75 tumour removals. Neither a clear distinction of tumour tissue against healthy brain tissue, nor differentiation of different tumour entities was possible on solely the Young’s modulus. Correlation between the stiffness grading of the surgeon and our measurements could be found.

**Conclusion:**

The mechanical behaviour of brain tumours given by the measured Young’s modulus corresponds well to the stiffness assessment of the neurosurgeon and can be a great tool for further information on mechanical characteristics of brain tumour tissue. Nevertheless, our findings imply that the information gained through indentation is limited.

**Supplementary Information:**

The online version contains supplementary material available at 10.1007/s00701-024-06218-4.

## Introduction

In the context of neurosurgical tumour operations on the central nervous system (CNS), the primary goal is to complete tumour resection without causing harm to the patient. Achieving this primary goal has immediate impacts on the recurrence-free tumour interval, on the patient’s quality of life and the lifetime. In the framework of neurosurgical interventions on the CNS, various multimodal technologies such as neuronavigation, fluorescence dyes [20], intraoperative ultrasound [1], or intraoperative MRI [17] are employed to achieve these objectives. Newer methodological approaches have also gained increasing prominence in the operating room, such as ultrasound elastography [18], thermography [14], impedance spectroscopy [16], optical coherence tomography [22, 24], Raman spectroscopy [12] and confocal laser microscopy [2, 3]. These last two technologies provide direct intraoperative information about tissue status corresponding to the gold standard of histological frozen section examination. Another important dimension in distinguishing healthy from diseased CNS tissue is the intraoperative tactile information obtained by the surgeon. Relying on haptic feedback is a physical skill of the neurosurgeon, developed through years of practice. Haptic feedback is composed of kinesthetic feedback, based on active pressure and muscle stimulation and tactile feedback, based on skin stimulation and passive pressure [19]. It can therefore help surgeons as an optional and additive source of information to palpate tumour tissue intraoperatively, both directly and via the instruments used, to distinguish it from healthy brain tissue [13]. One example for the relevance is evaluating objective haptic information for the integration into robotic systems [19]. In addition to this, objectifying intraoperative decision making in situ, real-time diagnostic tools would be of help and support for the surgeon. In particular in situations where a brain-shift occurs with respect to the installed neuronavigation system or where tumour tissue is not specific to fluorescent dyes or intraoperative ultrasound is unclear or unusable, tumour margin detection is difficult. Resulting tissue resection in healthy and potentially functional brain tissue can have severe postoperative consequences for the patient.

Budday et al. shows the complex and unique mechanical behaviour of healthy human and porcine brain tissue [5, 4, 7]. They developed mathematical models which describe tissue biomechanics. By doing indentation, shear, compression and relaxation experiments they determined the model parameters, Young’s modulus and stiffness [6]. To date, few studies have objectively quantified the mechanical properties of human brain tumours. The methods that have been proposed to objectively classify the consistency of tissue samples have mainly been tested on animals or fixed or frozen samples of human tumours, which can alter the elastic properties of the tissue [11]. A significant limitation of studies dealing with human brain tumours is that they often focus on a single or few tumour types and a limited number of samples on which a multitude of measurements are performed. [8, 10, 21] Furthermore, tumours were sometimes characterised using moduli other than the Young’s modulus or stiffness, making it difficult to compare with well-researched healthy tissue.

The aim of this work is to investigate an objective and reproducible method for measuring and characterising the mechanical properties of brain tumours and freshly excised brain tumour samples for comparison. Given the limitations of other studies in terms of patient numbers and tumour types, our approach allows us to provide an overview of the most common types of tumour, with ex-vivo measurements on fresh samples, to compare among each other and with healthy tissue. This approach could facilitate a more comprehensive understanding of the structural tissue properties of brain tumours and the distinctions between them and healthy tissue. Furthermore this could help to develop devices for intraoperative decision making. We derived Young’s modulus for the three most frequently diagnosed tumour entities: glioma, metastasis and meningioma, as well as healthy brain tissue.

## Methods

### Patients

**Fig. 1:**
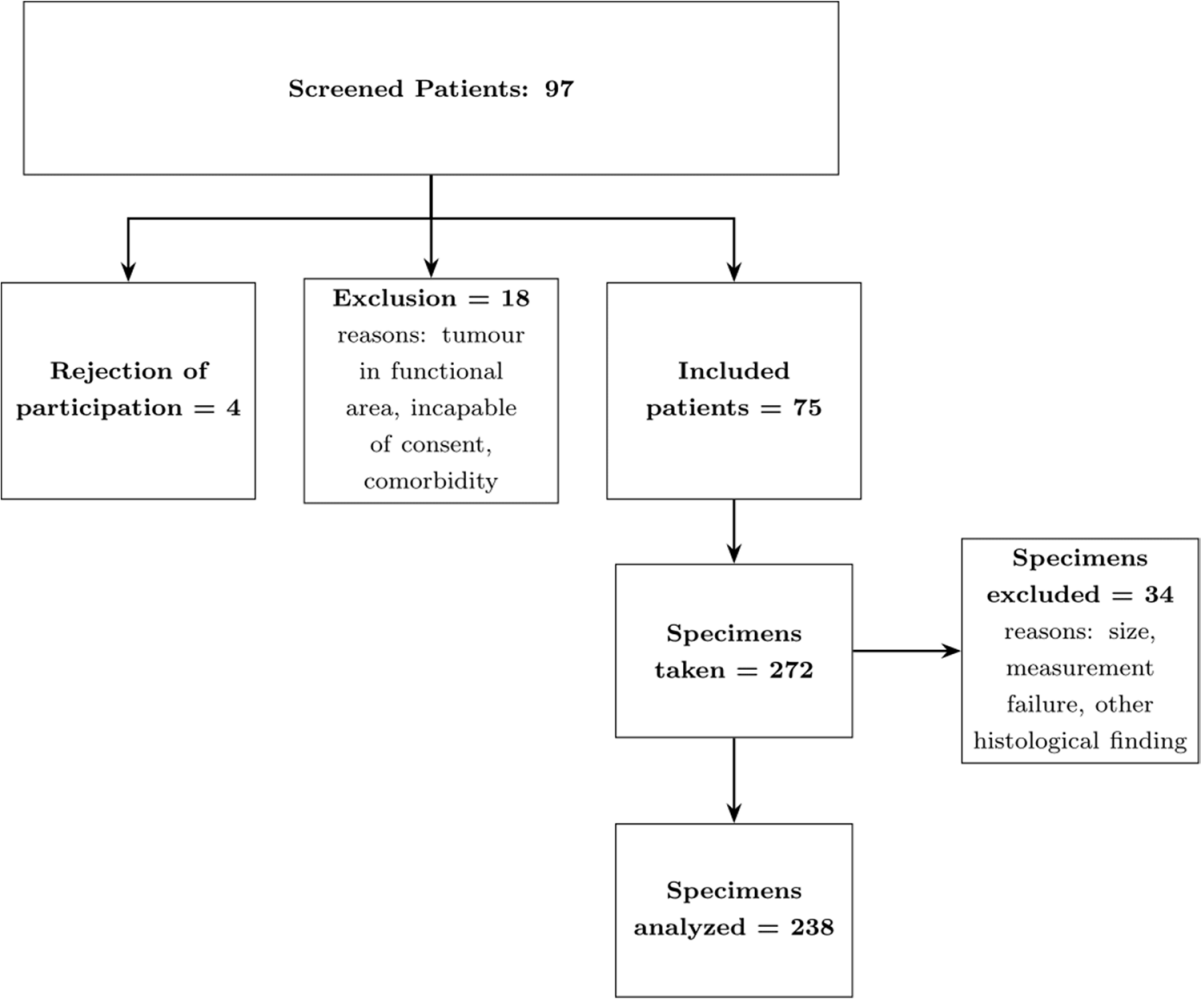
Flow chart of patient recruitment and sample acquisition. As some specimens were excluded for various reasons, the indentation measurements of 73 instead of the initial 75 surgeries were taken into account

Patients were recruited as part of regular clinical practice. Between November 2019 and February 2022, patients who underwent surgical removal of a brain tumour were screened. Inclusion criteria encompassed patients who were over 18 years old, able to provide consent, and had supratentorial tumours that were not situated in functional areas of the brain. The study excluded patients who were pregnant or had coagulation disorders, were taking blood thinners, or had serious comorbidities. The patient recruitment and sample acquisition is shown in Fig. 1. All patients underwent standard treatment after their tumour resection in accordance with current international neurosurgical guidelines. Afterwards, the study patients underwent an extensive screening for complications, and no complications were found to be associated with the study procedures. All patients were given detailed information about the study and given their written informed consent. All experiments were in agreement with the local ethics committee (Ethics Committee University of Luebeck, AZ 19–319) and performed according to the Declaration of Helsinki. Detailed patient information is shown in Table 1.

**Table 1:**
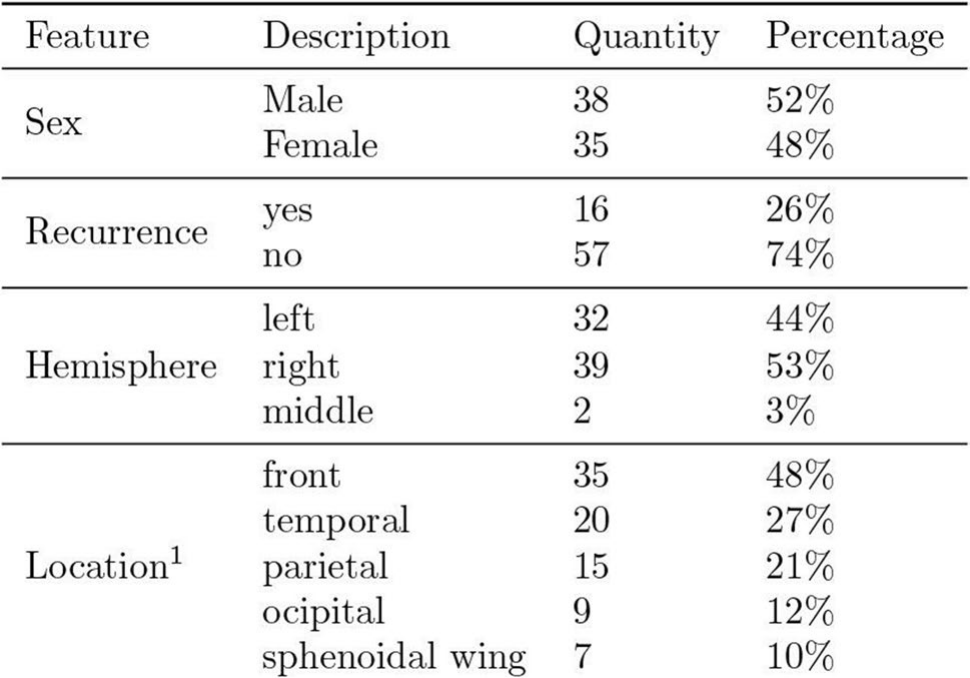
Patients characteristics

^1^ multiple selections possible

### Handling of tissue samples

Up to five tumour samples were extracted per surgery. Additionally during some surgeries we had access to healthy human brain tissue which was removed in order to reach the tumour, we extracted 30 healthy brain samples in total. All samples were collected using a 5 mm grasping forceps. At 2–8 locations on each biopsy, mechanical indentation measurements were performed within a five minute frame after sample collection. Our previous systematic experiments have shown that changes in tissue due to dehydration and autolysis processes, occur after 20 min, in two hours the Young’s modulus of the tissue increases by five times and after 24 h by a factor of 500. To avoid these processes, measurements were taken within five minutes of sample collection to preserve the original consistency of the tissue. Throughout the whole procedure, the sample was kept at room temperature (20°C). All samples were subsequently preserved in formalin, examined histopathologically and labelled by a neuropathologist. The label corresponds to the tumour type, there is no differentiation based on the percentage of tumour cells. In addition, the consistency of the tumour samples was labelled by the surgeon during removal. To grade tumour consistency, we used a numerical scale from 1 to 10, where 1 stands for very soft, deliquescent to fluid tissue and 10 for calcified tissue. Healthy tissue, which has a value of 4 on this scale, served as a reference. Additionally, the surgeon assessed whether the tumour consistency was homogeneous or not. This grading was used to evaluate the indentation measurement by correlating the mean value for the Young’s modulus of each tumour with the graded tumour stiffness of the surgeon.

### Test setup

**Fig. 2:**
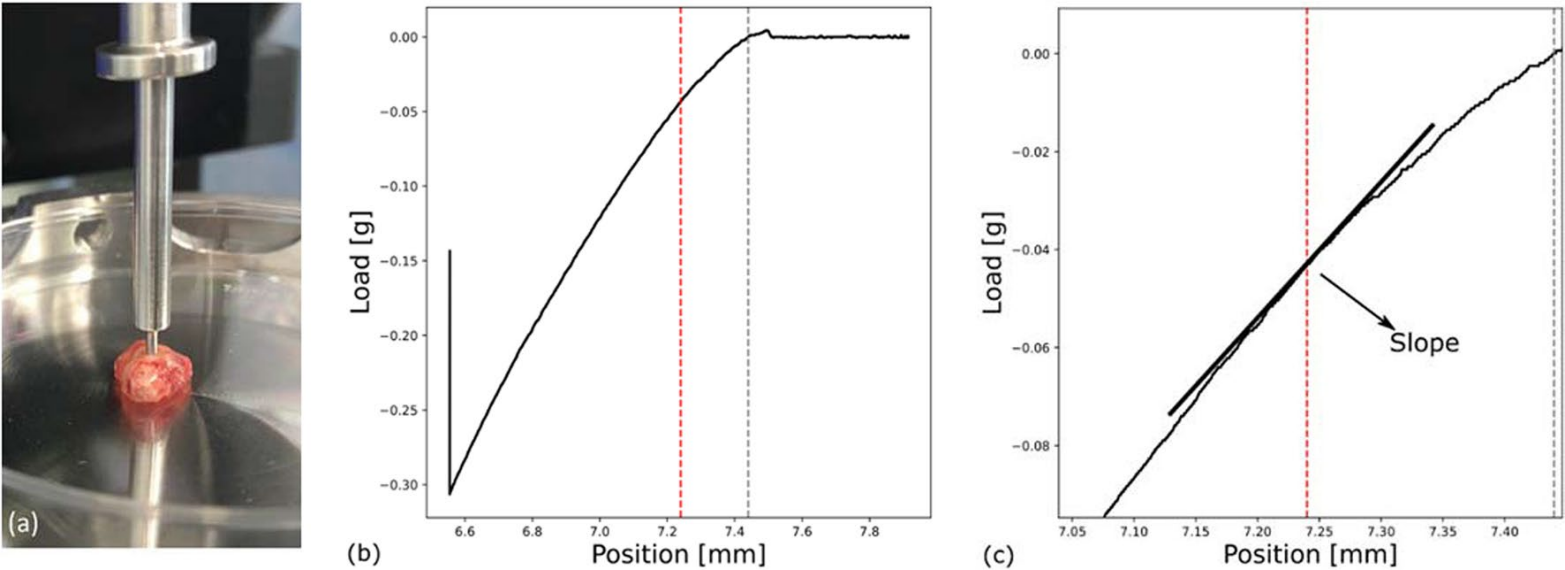
(**a**) Indentation measurement on a tissue biopsy from a brain tumour. (**b**) Example of measurement curve. The position indicates the vertical placement of the indenter. The grey line indicates the estimated tissue contact, the red line is at 200 µm indentation depth. (**c**) Enlarged section of the measurement curve to determine the slope at 200 µm indentation depth

A mechanical tester (Mach 1 v500c®, Biomomentum, Montreal, Canada) with a vertical stage enabled automated mechanical measurements with an indenter tip of $a=0.5{\text{mm}}$ in radius and a sensitive force sensor, with a load resolution down to 5 µN. The indenter was mounted below the force sensor as shown in Fig. 2(a). The vertical stage was set up to perform an automated linear movement with a speed of 0.1 mm per second, such that the indenter would move into the tissue with increasing force on the sensor. Measurements from samples outside the thickness range 2–10 mm were discarded to avoid size-effects. Furthermore, measurement data was discarded for further evaluation were the indenter had pierced the tissue in rare cases.

### Data evaluation

Each indentation measurement resulted in a force-indentation diagram as shown in Fig. 2(b). In order to enable comparison of different biopsies we used the same routine. We evaluated the measurement at an indentation of 200 µm by determining the slope S in the force-indentation diagram Fig. 2(c). Thus, the Young’s modulus was estimated from the slope of the curve in the linear region. As only samples with a thickness of more than 2 mm were used, this means that no sample were compressed by more than 10 percent of its original thickness at the indentation of evaluation. We calculated the respective Young’s modulus for each indentation measurement with the equation from Zhang et al. [25]:

1 $$E=\begin{array}{cc}\frac{\partial P}{\partial w}& \frac{\left(1-{v}^{2}\right)}{\kappa \left(a/n\right)}\end{array}$$

We can approximate $\frac{\partial P}{\partial w}=S$, were $S$ is the slope of the curve. Together with the assumption of incompressibilty for the Poisson ratio $v=0.5$ this leads to a simplified form for the Young’s modulus:

2 $$E=\begin{array}{cc}\frac{3}{4}& \begin{array}{c}S\\ \underset{\kappa \left(a/h\right)}{\to }\end{array}\end{array}$$

In this equation, h is the sample height and $a=0.5\text{mm}$ is the indenter radius. The numerical function $\kappa \left(a/h\right)$ is a correction term proposed by Hayes et al. [15]. Due to non-linearity, there is not only one defined Young’s modulus. The Young’s modulus of a sample was calculated using the mean of all measurements on this sample.

### Statistical analysis

The statistical analysis was conducted using the open-source statistical software R (version 4.3.2 binary for macOS). The Spearman’s rank coefficient ρ was utilised to examine the correlation between the Young’s modulus and the tumour stiffness grading as determined by the surgeon. In order to achieve a more accurate assessment, tumours labelled as inhomogeneous by the surgeon were excluded. For tumours that were labelled as homogeneous, the mean Young’s modulus over all samples was calculated, to ensure having a single grading and modulus for each tumour. Tumours labelled as inhomogeneous were still included in the evaluation of the Young’s modulus and only omitted for the correlation with the surgeon's stiffness grading.

## Results

**Fig. 3:**
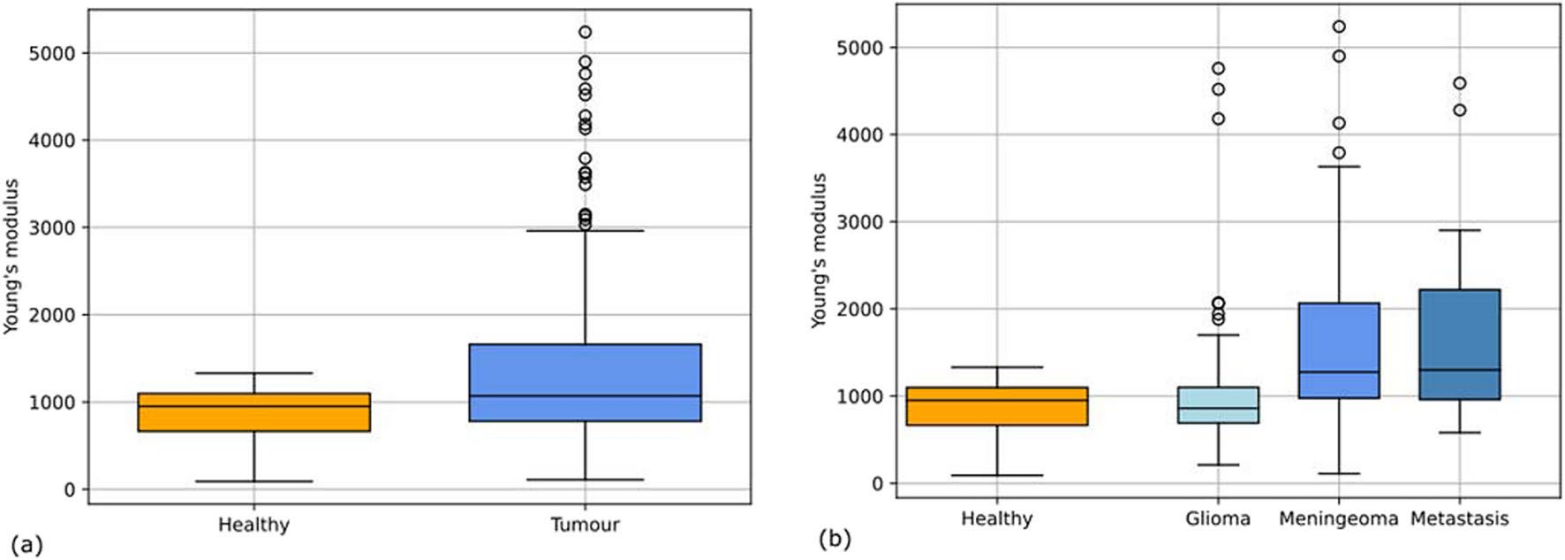
Distribution of elastic measurements of (**a**) healthy tissue against all tumour entities together and (**b**) all tumour entities separately

Over a period of two and a half years we examined 238 samples of 75 tumour removals and performed 934 indentation measurements in total,including surgeries of 34 meningiomas, 9 metastases and 30 gliomas. Figure 3 shows the distribution of Young’s modulus results for the brain tumour entities and healthy tissue in this study, more detailed data can be viewed in Table 2. The average Young’s modulus of healthy brain tissue is 866 Pa ± 279 Pa across measurements. The mean of meningiomas 1650 Pa ± 1007 Pa and metastasis 1682 Pa ± 1086 Pa are very similar, the mean of the Young’s modulus of gliomas 1038 Pa ± 732 Pa is slightly lower. In general, meningiomas and metastasis have a larger Young’s modulus and are stiffer than healthy brain tissue and gliomas. While the median of glioma tissue is slightly lower, the average is slightly firmer than the healthy tissue. Glioma tissue is the second largest sample group analysed in this study and has the strongest overlap with healthy brain tissue, with a slightly higher mean value.

**Table 2:**
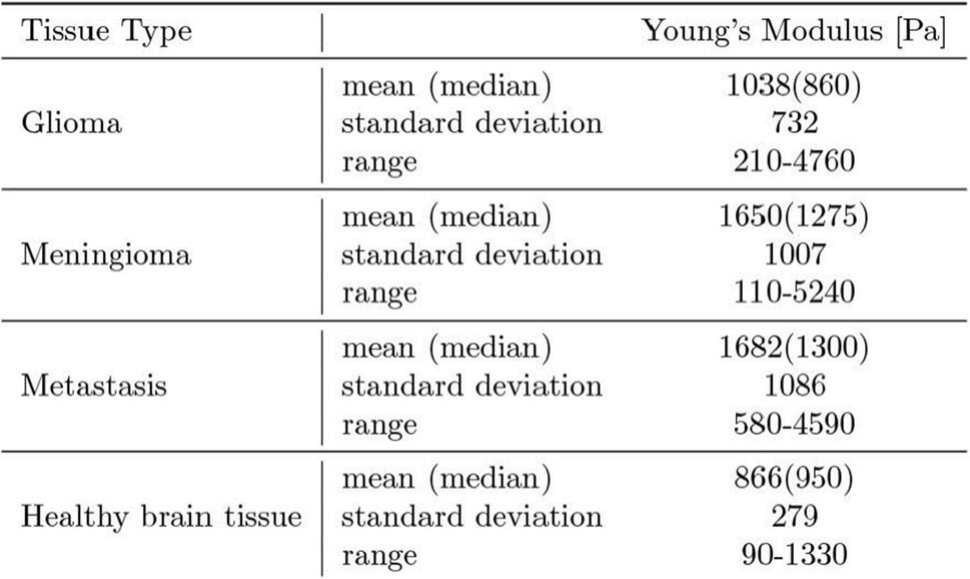
Young’s modulus measurement results

**Fig. 4:**
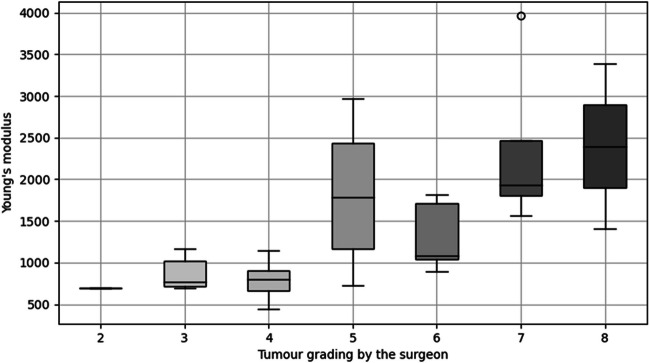
Correlation between the tumour stiffness grading by the surgeon and the measured mean Young’s modulus. A strong correlation (*ρ* = 0.67, p < 0.0001 Spearman’s rank coefficient) can be seen between both properties

We also examined the correlation between the stiffness grading by the surgeon during sample extraction and the Young’s modulus. The comparison can be seen in Fig. 4, there is a strong correlation (*ρ* = 0.67, p < 0.0001 Spearman’s rank coefficient) between both assessments. Especially at grading 4, where the tumour should have the same consistency as healthy tissue, the Young’s modulus of the tumour samples are in the same range as the healthy brain tissue samples.

## Discussion

The information provided by the haptic feedback is important for the neurosurgeon to distinguish between healthy and tumour tissue. With the advent of robotic surgical tools, methods to objectively measure this feedback are becoming increasingly important [19].

Budday et al. have developed mathematical models that describe the biomechanics of tissue. By performing indentation, shear, compression and relaxation experiments, they determine the model parameters [6]. In their recent review article [7] they summarise important facts about healthy human and porcine brain tissue: brain tissue is ultrasoft, highly fragile, biphasic, highly heterogeneous [23] and not particularly anisotropic. In their studies, where they used indentation measurements to show that the stiffness of healthy human brain tissue is characterised by a Young’s modulus of about 1400 Pa in grey matter and about 1900 Pa in white matter [5]. It should be noted that Budday et al.’s measurements were taken up to 48 h apart, whereas ours were taken immediately after collection. Dehydration of the tissue and autolysis processes can lead to stiffening of the tissue, which explains our comparatively low results for the Young’s modulus of healthy brain tissue 866 Pa ± 279 Pa.

Although recent studies on the mechanical properties of tumourous brain tissue have sometimes used different indentation devices and mathematical models, the results can still be compared based on the distribution of Young’s modulus or stiffness. We have shown that all three tumour types are highly heterogeneous, both within some samples and, more importantly, across all samples. This corresponds to results from different studies [8, 10, 21]. The most recent study by Černý et al. [8] also shows the heterogeneity among their meningioma samples *n* = 5. Our large sample number underlines these results. While being the second largest sample group analysed in this study, glioma tissue has the strongest overlap with healthy brain tissue. This goes well with the outcomes of Cie´sluk et al. [2]. Their data shows the strong inhomogeneity and slightly higher stiffness of glioblastoma tissue compared to healthy tissue, which fits previous research [9]. Our analysis confirms this, even though the difference is much less pronounced than expected, which can be partially explained by the ratio of number of samples and indentation measurements per sample. From this we draw, that a small quantity of two to eight measurements per sample can be sufficient for an overview over the heterogeneity and stiffness of tumour tissue. However, for more detailed information, more measurements across the sample may be useful.

We also compared our measured Young’s modulus to the stiffness grading by the surgeon during sample extraction. Overall, they were in good agreement. The outliers along this correlation could have different causes. One reason could be that different surgeons operated on the tumours and each surgeon might use a slightly different grading. It is also possible that the tumour grading is based on more dimensions of the surgeon’s haptic feedback, which means that Young’s modulus alone can only reflect part of this information.

Overall our analysis shows neither a clear distinction of tumour against healthy brain tissue, nor differentiation of different tumour entities solely by Young’s modulus is directly possible. It should be noted that we also looked at a comparatively small number of patients and samples of the three tumour entities mentioned. Since these results already show a wide distribution of Young’s modulus for each tumour entity, more measurements would not lead to a better differentiation of the tissue types.

## Conclusion

In this study, a direct intraoperative indentation measurement on human brain tissue was performed and analysed with regard to a possible distinction of different tumour entities. We were able to show that indentation is an objective and reproducible method for measuring and characterising the Young’s modulus of brain tumour tissue. Previous findings on healthy tissue were confirmed and the existing results for brain tumour tissue were expanded with a greater quantity of samples. In regards of tumour distinction the initial analyses of 238 samples show that the Young’s modulus alone is not sufficient to physically map and quantify the complexity of the mechanical properties of brain tumour tissue. The intraoperative haptic feedback available to an experienced surgeon contains more information than solely the elasticity, defined by the Young’s modulus, we examined in this study. The largest problem is the strong overlap in the softer regions of Young’s modulus, thus, this approach to classify tumour entities is limited. However, as we have demonstrated, mechanical indentation remains a promising method with the potential for further examination of mechanical properties.

## Supplementary Information

Below is the link to the electronic supplementary material.

Supplementary file1 (XLSX 10 KB)

## Data Availability

The data can be accessed by contacting the corresponding author.
